# Aspirin Inhibits Colorectal Cancer via the TIGIT-BCL2-BAX pathway in T Cells

**DOI:** 10.7150/ijms.98343

**Published:** 2024-08-01

**Authors:** Jiayu Liu, Qiunan Zhou, Kai Meng, Xiaojuan Yang, Bin Ma, Chunxia Su, Xiangguo Duan

**Affiliations:** 1 School of Inspection, Ningxia Medical University, Yinchuan 750004, China.; 2 Department of Pathogen Biology and Immunology, School of Basic Medical Science, Ningxia Medical University, Yinchuan 750004, China.; 3 Department of Oncology Surgery, The First People's Hospital of Yinchuan, Yinchuan 750004, China.; 4 The First School of Clinical Medicine, Ningxia Medical University, Yinchuan 750004, China.; 5 People's Hospital of Ningxia Hui Autonomous Region, Yinchuan 750004, China.; 6 Traditional Chinese Medicine Hospital of Ningxia Medical University, Yinchuan 750004, China.

**Keywords:** aspirin, CD155, CD226, colorectal cancer, TIGIT.

## Abstract

The T cell immunoglobulin and ITAM domain (TIGIT) is a recently discovered synergistic co-suppressor molecule that plays an important role in immune response and tumor immune escape in the context of cancer. Importantly, CD155 acts as a receptor for TIGIT, and CD155 signaling to immune cells is mediated through interactions with the co-stimulatory immune receptor CD226 (DNAM-1) and the inhibitory checkpoint receptors TIGIT and CD96. Aspirin (ASA) has been shown to reduce the growth and survival of colorectal cancer (CRC) cells, but the immunological mechanisms involved have not been sufficiently elucidated. In the present study the effects of aspirin on CRC in mice and on Jurkat cells were investigated. Aspirin may suppress the expression of TIGIT on T cells and Regulatory T cells (Tregs) and inhibit T cell viability, and therefore induce tumor cell apoptosis. TIGIT is expressed at higher levels on infiltrating lymphocytes within CRC tumor tissue than adjacent. Further, aspirin could inhibit Jurkat cell proliferation and induce apoptosis via downregulation of TIGIT expression and the anti-apoptosis B cell lymphoma 2 (BCL2) protein and upregulation of BCL2-associated X protein (BAX) expression. The present study suggests that aspirin can inhibit specific aspects of T cell function by reducing interleukin-10 and transforming growth factor-β1 secretion via the TIGIT-BCL2-BAX signaling pathway, resulting in improved effector T cell function that inhibits tumor progression.

## Introduction

Colorectal cancer (CRC) is the third most common malignant tumor worldwide and the second leading cause of cancer-related deaths in 2020 1. Approximately 1.9 million new cases of colorectal cancer occurred globally, accounting for 10.7% of all new cancer cases 2. Aspirin (ASA) is an antipyretic analgesic and anti-inflammatory drug can prevent certain cancers including colorectal cancer 3, 4. US and international guidelines recommended aspirin for the primary prevention of CRC in specific populations 3. Aspirin can reportedly exert antitumor effects by blocking AMPK/SIRT3-mediated glycolysis 5 and inhibiting Blimp1, activating ATF4/CHOP pathway 6. Specific interactions between aspirin and T cells in CRC remain unclear. Therefore, investigation of the molecular basis of the effects of aspirin is urgently needed.

The tumor microenvironment is crucial in the development of tumors, promoting the proliferation, survival, and migration of tumor cells. It is composed of immune cells, tumor cells, stromal cells, and extracellular matrix 7. Immune cell-mediated tumor evasion is one of the key mechanisms involved in the progression and survival of malignant cells 8. T cell activation requires dual signaling involving the recognition of antigenic tumor peptides by T cell receptors, along with costimulatory signals. T cell immunoglobulin and immunoreceptor tyrosine-based inhibition motif domain (TIGIT) is one of the related co stimulatory molecules. The TIGIT receptor is mainly expressed on natural killer cells, regulatory T cells (Tregs), CD8+ T cells, and CD4+ T cells 9. TIGIT-expressing forkhead box P3 (FOXP3)+ T cells have been characterized as a highly functional Treg subset that selectively suppresses Th1 and Th17 responses^10^, ^11^. CD155 is a member of the nectin-like family of proteins that has recently been investigated as a potential target in immunotherapy that enhances anti-tumor responses 12. It is highly upregulated on tumor cells across multiple cancer types and has been associated with worse patient outcomes. Besides, CD155 has now been implicated in immune regulation. CD155, CD226, inhibitory checkpoint receptors TIGIT and CD96 are differentially regulated at the cell surface on T cells and NK cells 13. TIGIT and CD155 have higher affinity than CD226^14^. Engagement of TIGIT with CD155 promotes interleukin (IL)-10 while simultaneously reducing IL-12 production by dendritic cells, leading to reduced T cell activation *in vitro* 15, 16.

In the current study the expression levels of different factors that function as immune checkpoints and ligands in the CRC tumor environment were investigated. The effects of aspirin on the biological functions of Jurkat cells and Tregs were assessed, as was the extent to which T cell function could be inhibited by aspirin via the TIGIT pathway. Levels of B cell lymphoma 2 (BCL2), BCL2-associated X protein (BAX), and TIGIT expression were investigated after aspirin treatment. Lastly, biological changes in T cells that were influenced by the TIGIT signaling pathway were assessed after aspirin administration.

## Materials and Methods

Aspirin was purchased from American Sigma Company and dissolved in anhydrous ethanol just before use.

### Inclusion and exclusion criteria of clinical samples

The tumor tissues and paracancerous tissues of CRC patients were collected: colorectal cancer and paracancerous wax tissue specimens of CRC patients were obtained from 5 patients who underwent surgical resection in the Department of Oncology Surgery, The First People's Hospital of Yinchuan, the Second Clinical Medical College of Ningxia Medical University.

The inclusion criteria were as follows: all patients underwent resection of colon cancer for the first time, had not received anti-tumor treatment before operation, and were diagnosed as CRC by pathology after operation. Exclusion criteria: Patients with a history of hypertension, diabetes and other chronic diseases; Patients with history of other tumors, Hepatitis B, tuberculosis.

### Cell culture

Jurkat cells were purchased from Procell (Wuhan, China), and cultured in RPMI1640 (Gibco) containing 10% fetal bovine serum (PAN Biotech).

### Immunohistochemistry analysis

CRC tissue samples and paracancerous tissue samples were fixed with 4% paraformaldehyde and embedded in paraffin, then cut into 4-µm-thick sections, incubated at about 60°C for 2 h, then subjected to immunohistochemical staining. The sections were deparaffinized, rehydrated and treated with 3% H_2_O_2_ to eliminate the activity of endogenous peroxidase. Antigen recovery was performed and blocked with 5% goat serum. After incubation with anti-TIGIT antibody, anti-CD226 antibody and anti-CD155 antibody (Abcam) overnight, the samples were incubated with the secondary antibodies at room temperature for 30 min, stained with diaminobenzidine for 90 s, then counterstained with hematoxylin for 4 min. Finally, the distribution and expression of the target antigen in the tissue were observed under a microscope.

### Cell viability

Jurkat cells were seeded into 96-well microplates (3.0 x 10^4^ per well) in a total volume of 200 µL, and cultured with various concentrations of aspirin for 24 h, 48 h, and 72 h. Cell Counting Kit-8 solution (BestBio, Shanghai, China) was then added to the cells at 10 µL per well, and they were incubated for a further 1 h at 37 °C. Optical density was measured at an absorbance of 450 nm.

### Apoptosis detection

The apoptosis of cells was detected by flow cytometry. Performed membrane associated protein V-fluorescein isothiocyanate (FITC)/propidium iodide dual staining assay according to the manufacturer's protocol (KeyGen BioTech, Nanjing, China). Washed the cells with phosphate buffered saline (PBS), then resuspended them in buffer and incubated with membrane associated protein V-FITC and propidium iodide in the dark for 15 minutes. Then flow cytometry was performed, and BD Accuri C6 software was used for analysis.

### Mouse experiments

Male C57BL/6N mice (age 6 weeks, bodyweight 18-22 g) were purchased from Beijing Huafukang Bioscience, Beijing, China (the male has better tolerance to drugs and could reduce unnecessary sacrifices in mice), and housed under specific pathogen-free conditions with food and water available *ad libitum*. The construction of a mouse CRC model induced by AOM/DSS (Azoxymeth/Extran Sulfate Sodium Salt).

The mice were randomly assigned to 3 groups, a control group (normal mice), an AD group (CRC mice without aspirin), an SA group (aspirin added to AIN-93G purified feed at 200 mg/kg), and an HA group (aspirin added to AIN-93G purified feed at 400 mg/kg), 6 mice per group. All experimental procedures were conducted in strict accordance with the operating procedures of the Animal Experimental Center of Ningxia Medical University, China (approval number 2014-005). All operations were performed with the aim of minimizing the suffering of the mice, attrition rate ≤ 20%.

Aspirin was purchased from SIGMA company in the United States, with a purity of ≥ 99.05%. According to previous research results, mice consume approximately 2.5g of feed per day, with a feed ratio of 200mg/kg added. That is, each mouse consumes 500ug of aspirin per day. According to this ratio, the mouse dose (mg/kg) is 9.01 × the dose of adult aspirin (100 mg)/adult body weight (60kg) is equivalent to an adult taking 80-110 mg of aspirin per day.

### Immune cell isolation

Mice were killed at the age of 16 weeks, and spleens were dissected from surrounding tissues and processed for flow cytometry analysis. Spleens were mechanically homogenized in PBS, then the resulting preparations were filtered through 300 mesh filters, superimposed on lymphocyte separating fluid at 2000 rpm for 20 min. Mononuclear cells thus obtained were transferred into fresh tubes then centrifuged at 1500 rpm for 10 min. Lastly, the cell concentration was adjusted to 1 x 10^6^/mL incubation flow cytometry antibody for flow detection.

### Flow cytometry

Cells were rinsed in PBS then incubated at 4 °C with the following four monoclonal antibodies simultaneously (BD Biosciences) prior to flow cytometry analysis: FITC-conjugated anti-CD4, phycoerythrin-conjugated anti-TIGIT, allophycocyanin-conjugated anti-CD25, peridinin-chlorophyll-protein-cyanine 5.5-conjugated anti-FOXP3. Single-stained control labeling was performed in parallel.

### Western blotting

After washing the cells twice with pre-cooling PBS, the cells were lysed using cell lysis buffer (KeyGen BioTech). The protein concentration was quantified using the KeyGen BioTech assay kit. 5 μ g of total protein was electrophorized on 10% sodium dodecyl sulfate polyacrylamide gel, and then transferred to the 0.22 μ m polyvinylidene fluoride membrane (Millipore, USA). These PVDF membranes were sealed in 5% fat free milk for 2 hours, and then incubated overnight with primary antibodies at 4 °C. After labeling the secondary antibody with horseradish peroxidase (Abbkine, China), the signal was detected using an enhanced chemiluminescence assay kit (Advancta, England). The obtained images were analyzed using ImageJ 6.0 software. The antibody against glyceraldehyde 3-phosphate dehydrogenase (GAPDH) was used as a control for whole cell lysates.

### Statistical analysis

Continuous variables are expressed as medians and ranges, or as means ± the standard deviation. Analysis of variance was used for statistical comparisons. All statistical analyses were performed using SPSS 23.0, and two-sided *p* values < 0.05 were deemed to indicate statistical significance.

## Results

### TIGIT, CD226, and CD155 expression on lymphocytes in CRC tissue and adjacent tissues

**Figure 1:**
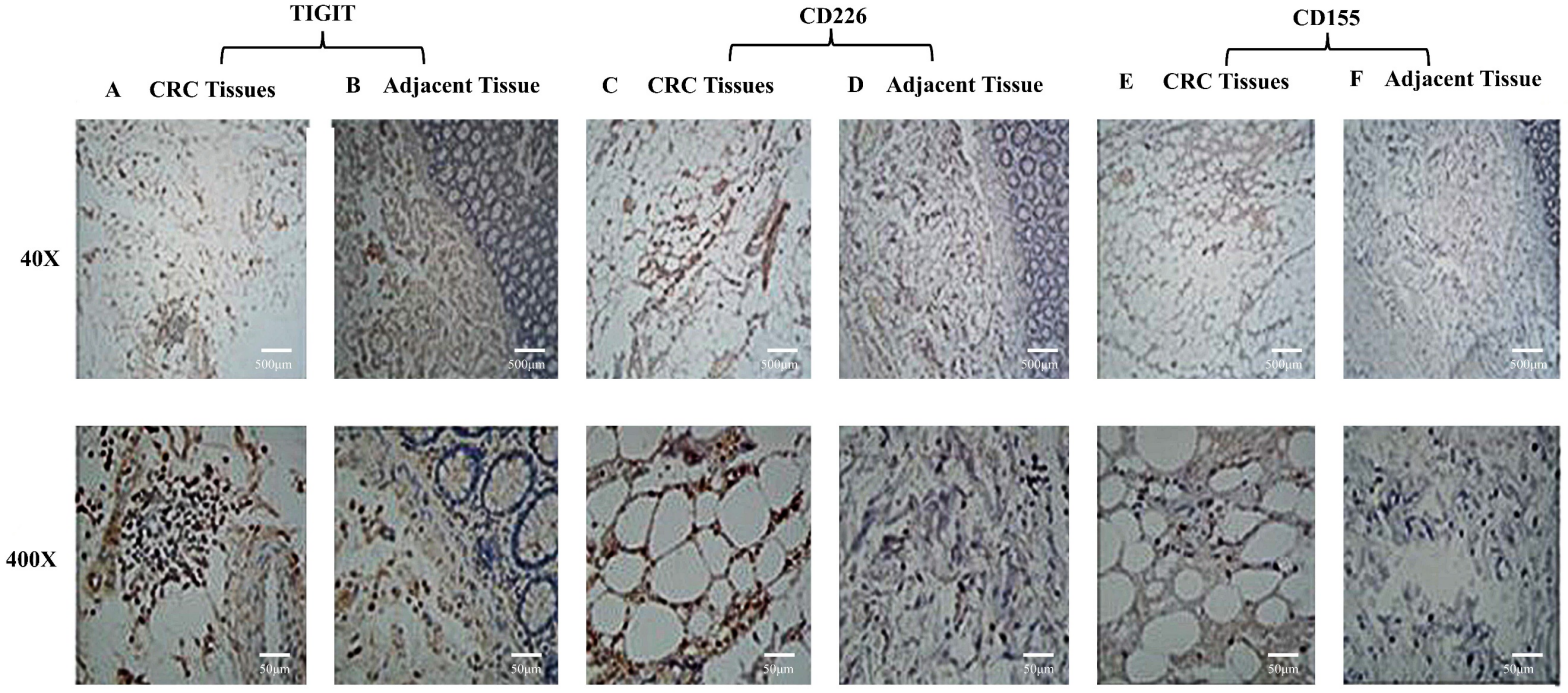
** TIGIT, CD226, and CD155 expression by lymphocytes in CRC tissue and adjacent tissue.** TIGIT and CD226 were expressed by lymphocytes in colorectal carcinomas. CD155 was not expressed by lymphocytes. **(A)** Colon adenocarcinoma tissue stained positively with polyclonal anti-TIGIT. **(B)** A section of adjacent tissue stained with an irrelevant polyclonal anti-TIGIT antibody (x40, x400). **(C)** Colon adenocarcinoma tissue stained positively with polyclonal anti-CD226 (x40, x400). **(D)** A section of adjacent tissue stained with an irrelevant polyclonal anti-CD226 antibody (x40, x400). **(E)** Colon adenocarcinoma tissue stained positively with polyclonal anti-CD155 (x40, x400). **(F)** A section of adjacent tissue stained with an irrelevant polyclonal anti-CD155 antibody (x40, x400). Scale bar, 500 μm and 50 μm.

TIGIT and CD226 expression were higher in lymphocytes in CRC tissues than adjacent, and they were mainly expressed in lymphocyte plasma membranes (Figure 1A-1D). CD155 expression was not detected on lymphocytes (Figure 1E, 1F). The results suggest that TIGIT and CD226 may compete for binding with CD155.

### Effects of aspirin on pathological changes in colon cancer mice

**Figure 2:**
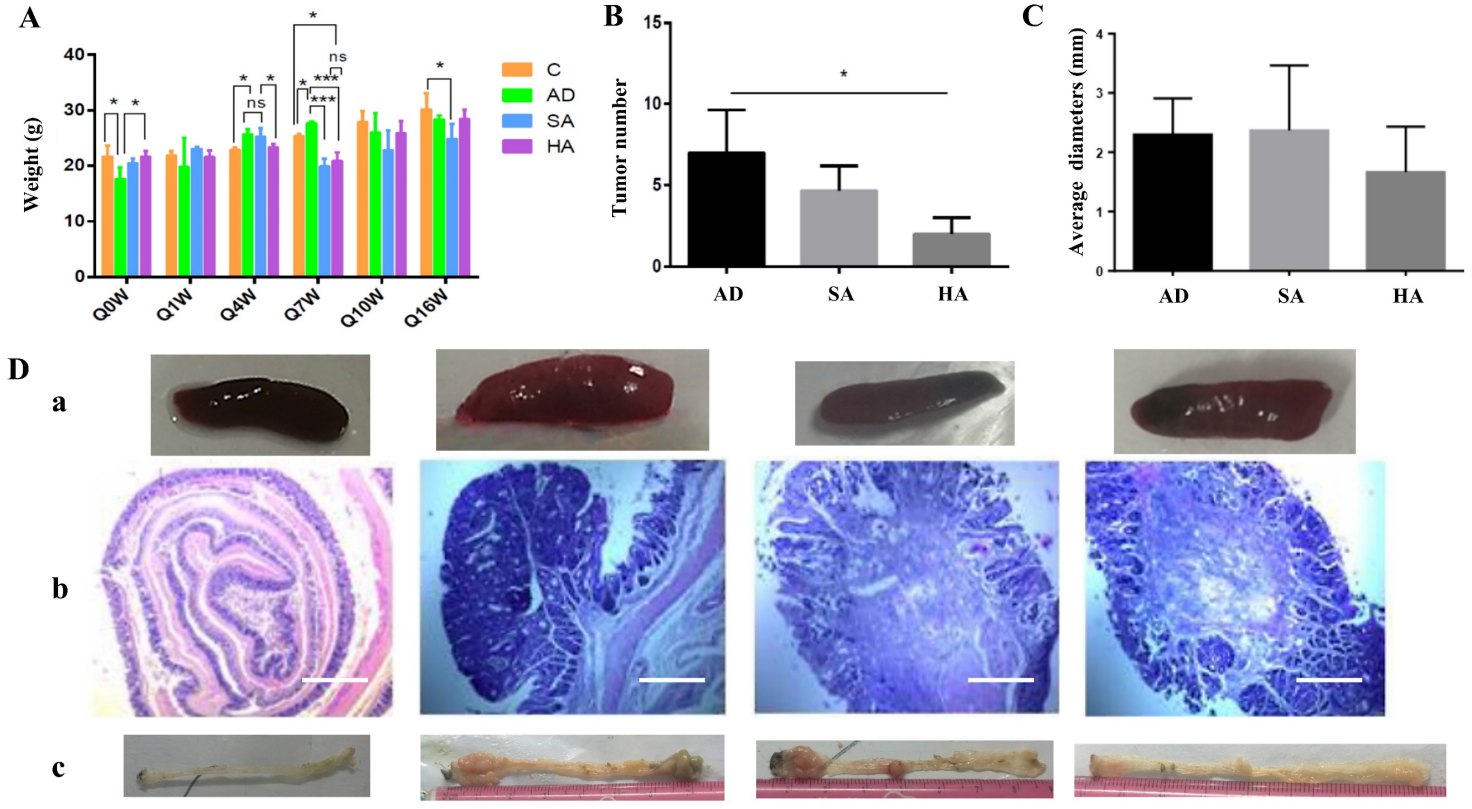
** Effects of aspirin on pathological changes in a murine CRC model. (A)** Bodyweights in CRC mice treated with aspirin, measured every 3 weeks. **(B, C)** Tumor numbers and tumor diameters after treatment with aspirin. **(Da)** Splenic changes. **(Db)** Hematoxylin-eosin staining depicting colorectal changes after treatment with aspirin (original magnification x200). Scale bar, 1000 μm. **(Dc)** Tumor number and tumor position. The results represent means ± the standard deviation. Control--normal mice, AD--CRC mice without aspirin, SA--low-dose aspirin added to feed, HA-- high-dose aspirin added to feed. *p < 0.05, **p < 0.01 in comparison with the control.

Mice were divided into control group (normal mice), an AD group (CRC mice without aspirin), an SA group (lower-dose aspirin added to feed), and an HA group (higher-dose aspirin added to feed). At the beginning, the weight of both Group C and Group HA was higher than that of Group AD, with statistical differences due to individual differences randomly assigned. Aspirin administration affected bodyweight in mice with colon cancer, especially at week 4 and week 7. However, from the overall picture, drug induced tumor formation and ASA prevention of tumors have no long-term effect on mouse body weight (Figure 2A). Tumor numbers were significantly lower in mice administered aspirin (Figure 2B). There were small differences in tumor diameter in each group (Figure 2C). The above results indicate that high-dose ASA (400mg/kg) can effectively inhibit tumors, but ASA has little effect on tumor diameter. Splenic changes in mice administered aspirin are shown in Figure 2Da. Hematoxylin and eosin staining further confirmed that aspirin could reduce the tumor burden in CRC mice (Figure 2Db).

Compared with other groups of mice, the size and morphology of the spleen in the normal group were normal. However, among the AD group, SA group, and HA group, the situation in the latter two groups improved compared to the former. The spleen was smaller and the tumor burden was relieved. Tumor numbers and locations are shown in Figure 2Dc. After dissecting the mice, it was found that the majority of tumors grew in the rectum near the anus, followed by the middle and lower segments of the colon. Tumors in the upper segment were rare. The AD group had significantly larger and more tumors, while the SA group had slightly fewer tumors. The HA group had significantly fewer tumors in terms of number and size.

### Low TIGIT expression in splenic lymphocytes in CRC mice

**Figure 3:**
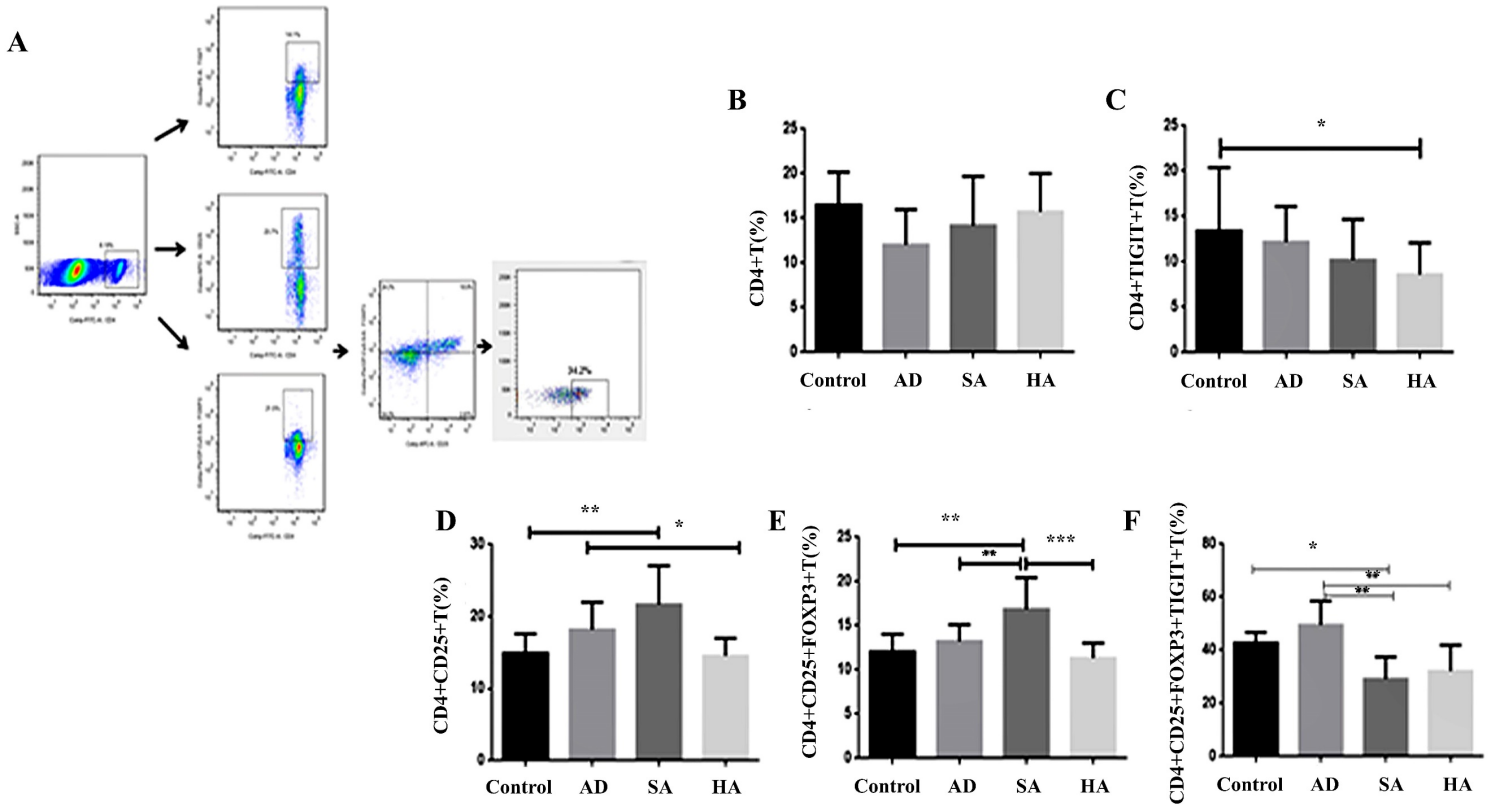
** Low TIGIT expression in splenic lymphocytes from CRC mice. (A)** Representative flow cytometry plots of Tregs as determined by TIGIT and chemokine receptor expression gated on CD4+ T cells in healthy controls (*n* = 5). **(B)** CD4+ T cells. **(C)** TIGIT expression on CD4+ T cells. **(D)** CD4+CD25+ T cells. **(E)** CD4+CD25+FOXP3+ T cells. **(F)** TIGIT expression on CD4+CD25+FOXP3+ T cells.

The results of flow cytometry analysis of TIGIT expression in gated CD4+ T cells and Treg cells from peripheral total blood mononuclear cells derived from CRC mice are shown in Figure 3A-3C. Levels of CD4+TIGIT+ T cells were 13.42% ± 6.96 in controls, 12.19% ± 3.90 in AD mice, 10.20% ± 4.47 in SA mice, and 8.58% ± 3.53 in HA mice (Figure 3C). Levels of CD4+CD25+ T cells were 14.88% ± 2.72 in controls, 18.16% ± 3.82 in AD mice, 21.64% ± 5.39 in SA mice, and 14.51% ± 2.50 in HA mice (Figure 3D). Levels of CD4+CD25+FOXP3+ T cells were 12.04% ± 2.02 in controls, 13.23% ± 1.89 in AD mice, 16.86% ± 3.60 in SA mice, and 11.32% ± 1.73 in HA mice (Figure 3E). Levels of the CD4+CD25+FOXP3+TIGIT+ T cells were 42.67% ± 4.07 in controls, 49.40% ± 9.09 in AD mice, 29.07% ± 8.38 in SA mice, and 32.12% ± 9.76 in HA mice (Figure 3F). Compared with the AD group the levels of CD4+CD25+ T cells, CD4+CD25+FOXP3+ T cells, and CD4+CD25+FOXP3+TIGIT+ T cells were lower in the HA group. Compared with the AD group the levels of CD4+CD25+ T cells, CD4+CD25+FOXP3+ T cells, and CD4+CD25+FOXP3+TIGIT+ T cells were lower in the HA group.

### Aspirin inhibited cell viability in Jurkat cells

**Figure 4:**
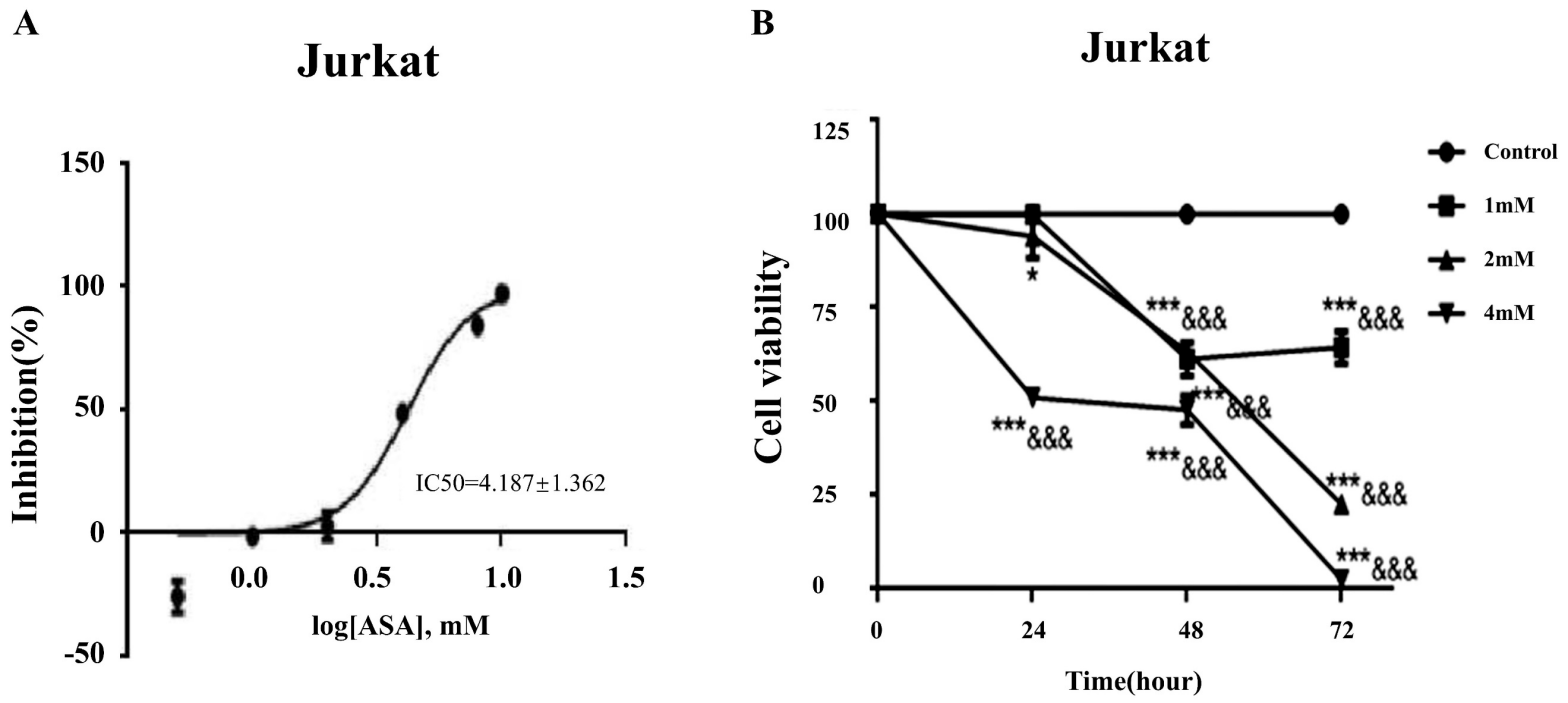
** Aspirin inhibited the viability of Jurkat cells. (A)** The IC50 curve of aspirin intervention Jurkat. **(B)** Viability of Jurkat cells treated with 1, 2, or 4mM concentrations of aspirin for 24, 48, and 72 h. *p < 0.05, **p < 0.01, ***p < 0.001 compared to the control at that same timepoint. &*p* < 0.05, &&*p* < 0.001, &&&*p* < 0.0001 compared to the control at 0 h.

The IC50 curve of aspirin intervention Jurkat showed that the IC50 was 4.187 ± 1.362Mm (Figure 4A). Cell counts in Jurkat cell cultures exposed to 1, 2, and 4mM concentrations of aspirin for 24, 48, and 72 h are shown in Figure 4B. After exposure to 4 mM for 24 h the viability of Jurkat cells was significantly reduced. When aspirin reached a sufficient concentration the inhibition of cell activity was time-dependent and concentration-dependent.

### Aspirin enhanced the rate of Jurkat cell apoptosis

**Figure 5:**
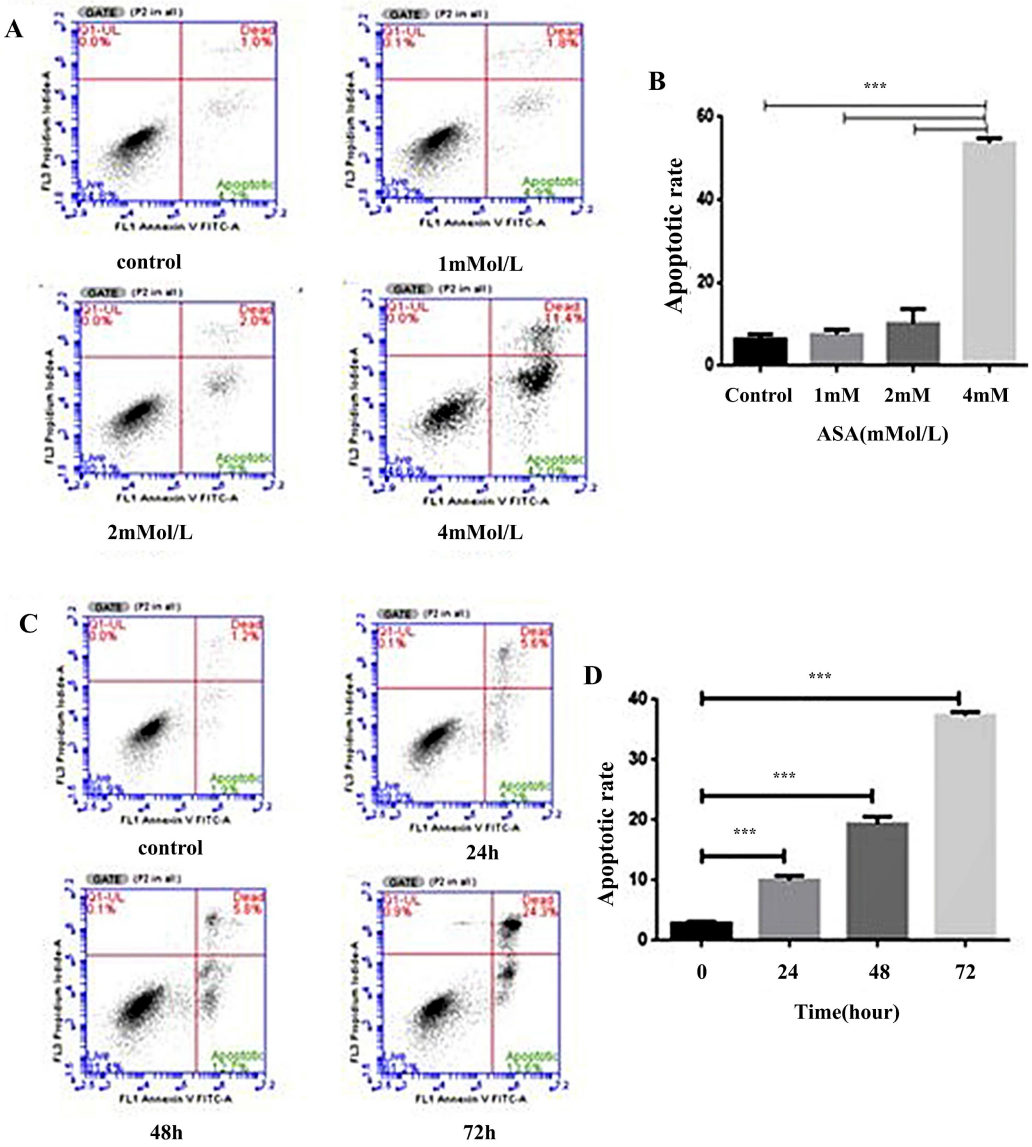
** Aspirin induced Jurkat cell apoptosis. (A, B)** Jurkat cells were stained with annexin-V-FITC and propidium iodide after exposure to 1, 2, and 4mM concentrations of aspirin for 48 h. **(C, D)** Jurkat cells were stained with annexin-V-FITC and propidium iodide after exposure to a 4 mM concentration of aspirin for 0, 24, 48, or 72 h. Data are indicative of three separate experiments. The statistical significance of the results was assessed using one-way analysis of variance followed by Fisher's least significant difference test. *p < 0.05, **p < 0.01, ***p < 0.001 compared to the control.

The effects of increased concentrations of aspirin on apoptosis in Jurkat cells are shown in Figure 5. Compared to the untreated control, aspirin induced a marginal to moderate increase in apoptosis (Figure 5A, 5B). At a concentration of 4 mM the amount of apoptosis was time-dependent (Figure 5C, 5D).

### Effects of aspirin on anti-apoptotic and pro-apoptotic proteins

**Figure 6:**
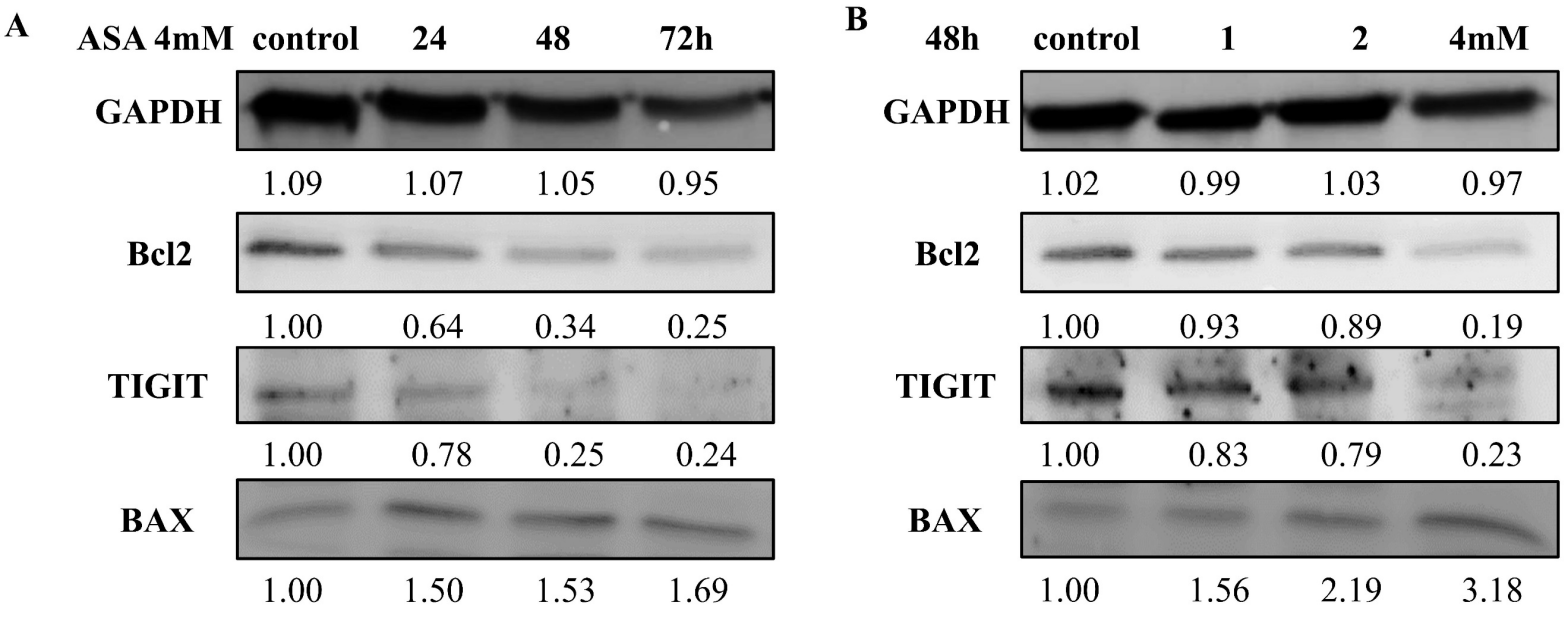
** Western blotting of TIGIT, BAX, and BCL2 expression by Jurkat cells after exposure to aspirin. (A)** Effects of exposure to 1, 2, and 4mM concentrations of aspirin for 48 h. **(B)** Effects of exposure to a 4 mM concentration of aspirin for 24, 48, and 72 h. An anti-GAPDH antibody was used as a loading control. *p < 0.05, **p < 0.01, ***p < 0.001 compared to the control.

Aspirin increased the expression of the pro-apoptotic protein BAX, and the increases were time-dependent (Figure 6A) and concentration-dependent (Figure 6B). At an aspirin concentration of 4 mM there was a time-dependent reduction in the expression of the anti-apoptotic protein BCL2 (Figure 6B). Aspirin had a slight concentration-dependent influence on the expression of the anti-proapoptotic protein BCL2 (Figure 6A). The results quantified by ImageJ 6.0 software (relative to the expression of GAPDH).

### Aspirin attenuated TIGIT expression in Jurkat cells

The effects of aspirin on the expression of TIGIT in Jurkat cells are shown in Figure 6. Aspirin reduced TIGIT expression, and the reductions were concentration-dependent (Figure 6A) and time-dependent (Figure 6B).

### Aspirin inhibited Jurkat cell transforming growth factor-β1 and IL-10 secretion

**Figure 7:**
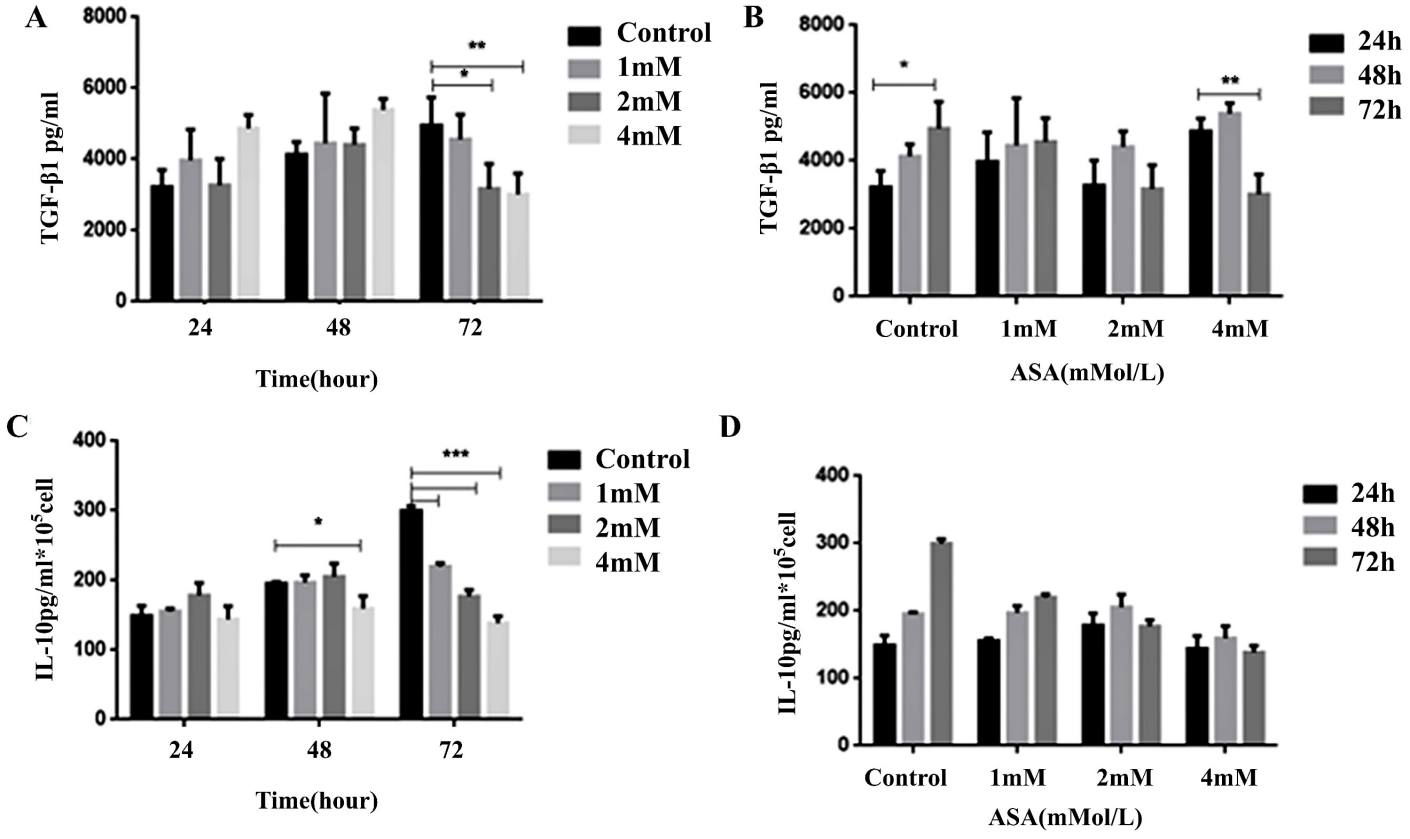
** Dynamic changes in cytokine levels in Jurkat cell culture supernatants.** Enzyme-linked immunosorbent assays were used to determine **(A, B)** TGF-β1 and **(C, D)** IL-10 levels in the supernatants of Jurkat cell cultures after exposure to 1, 2, or 4mM concentrations of aspirin for 24, 48, and 72 h. All data are expressed as means ± the standard deviation of three separate experiments. *p < 0.05, **p < 0.01, ***p < 0.001 compared to the control.

The results of enzyme-linked immunosorbent assays conducted to determine levels of transforming growth factor (TGF)-β1 and IL-10 in the supernatant of Jurkat cell cultures after exposure to aspirin are shown in Figure 7. There were significant concentration-dependent reductions in both TGF-β1 secretion and IL-10 secretion.

### Schematic representation of the effects of aspirin on Tregs and Jurkat cells

**Figure 8:**
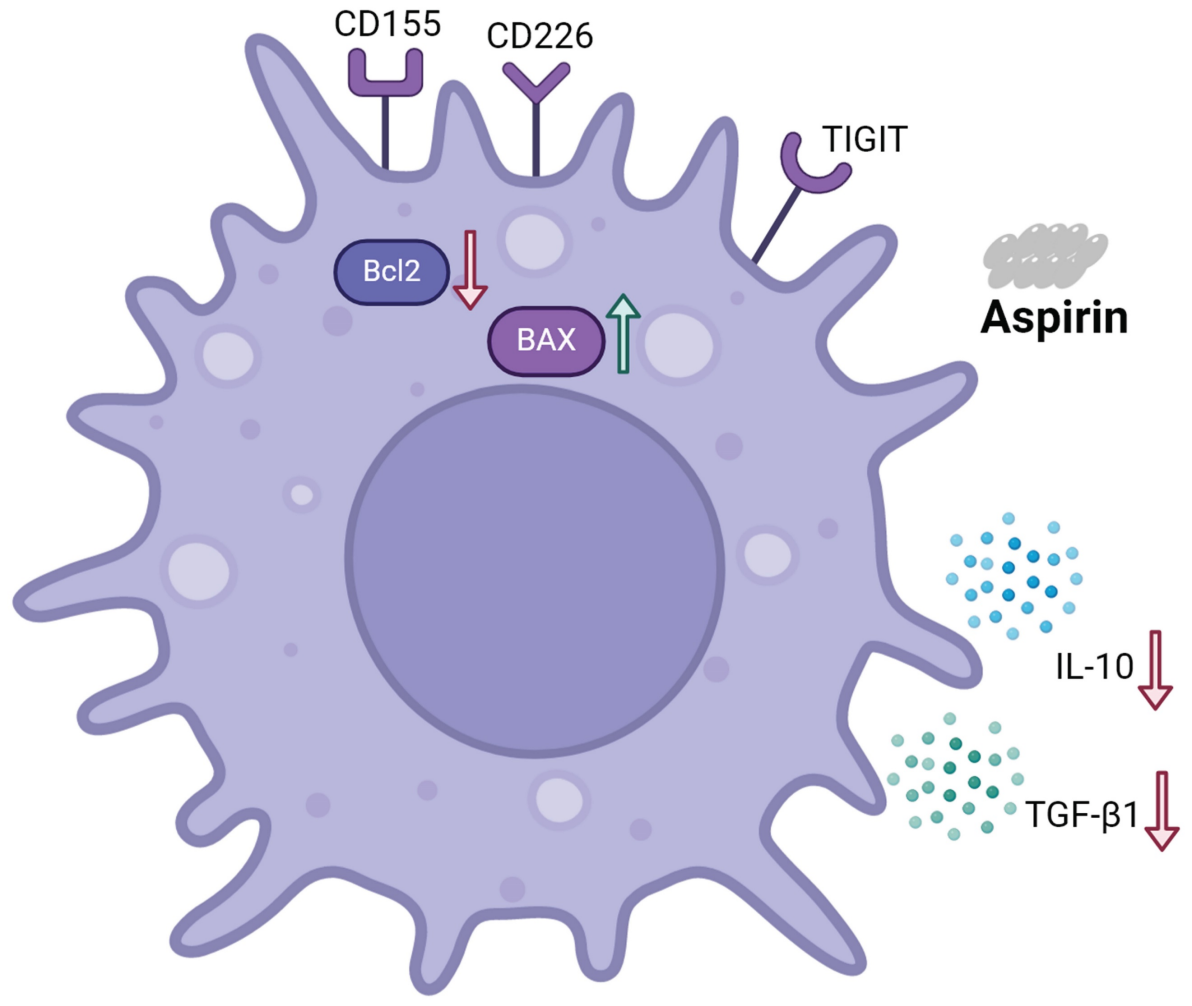
** Schematic representation of the effects of aspirin on Tregs and Jurkat cells.** Aspirin induced Jurkat cell apoptosis by upregulating the expression of pro-apoptotic protein BAX and downregulating the expression of anti-apoptotic protein BCL2. Aspirin can induce apoptosis of Tregs through the TIGIT pathway and inhibit the secretion of TGF-β1 and IL-10 by Jurkat cells to improve effector T cell function and ultimately inhibit tumors.

As shown in Figure 8, aspirin induced Jurkat cell apoptosis. Expression of the pro-apoptotic protein BAX was upregulated. Expression of the anti-apoptotic protein BCL2 was downregulated. Aspirin can induce apoptosis of Tregs via the TIGIT pathway, improving effector T cell function, ultimately resulting in tumor inhibition. Jurkat cell secretion of TGF-β1 and IL-10 were inhibited by aspirin.

## Discussion

In the present study aspirin induced apoptosis in CRC cells. TIGIT is mainly expressed on natural killer cells, Tregs, and CD8+ and CD4+ T cells. CD4+CD25+FOXP3+ Tregs inhibit immune responses by influencing other T cell subsets 17. Treg infiltration in tumors, scored as FOXP3+ or CD4+ /CD25+ /FOXP3+ (triple-positive) cells, was strongly correlated to the overall amount of CD3+ and CD8+T cells 18. In previous studies, high densities of infiltrating Tregs were associated with adverse prognoses in patients with some cancer types 19, 20. TGF-β1 and IL-10 are mainly secreted by Tregs and linked to its biology 21, 22. IL-10 is currently considered an immunosuppressive factor that regulates cell growth and differentiation, participates in inflammatory and immune responses. It plays an important role in cancer, infection, and organ transplantation 23. TGF- β 1 can inhibit immune function, regulate cell growth and differentiation, inhibit proliferation of various tumor cells, and activate cell apoptosis 21, 24.

In the current study aspirin was associated with reduced expression of the inhibitory molecule TIGIT on Tregs and Jurkat cells, resulting in reduced cell viability and the induction of apoptosis. Based on these observations we hypothesize that aspirin may influence tumor immunity and engage in the tumor environment. In the present study TIGIT and CD226 were expressed at higher levels by lymphocytes infiltrating CRC tumor tissues than adjacent, but the expression of CD155 was low or absent. However, due to the limited sample size, there are certain limitations, which can be further expanded in the future. CD155 was highly expressed in colorectal carcinomas 25. TIGIT binds to CD155 with higher affinity than the costimulatory CD155-binding receptor CD226^12^. Interestingly this is consistent with the results of the present study, and suggests that TIGIT bound to CD155 resulting in the activation of inhibitory signaling that reduced effector T cell function. Lymphocytes participate in mediating immune escape in tumor cells within the tumor microenvironment. TIGIT and CD226 can compete for binding with CD155, and induce different immune responses in the tumor environment. We hypothesize that TIGIT is involved in this process during tumorigenesis.

Considerable evidence demonstrates that regular use of aspirin is effective in reducing the risk for precancerous colorectal neoplasia and colorectal cancer 3, 26. Aspirin has been shown to exert direct antitumor effects by blocking AMPK/SIRT3-mediated glycolysis 5 and inhibiting Blimp1, activating ATF4/CHOP pathway 6. In this study aspirin induced apoptosis of Jurkat cells, BAX was upregulated in a concentration-dependent manner, BCL2 was downregulated, and TIGIT expression was significantly reduced in a concentration-dependent manner. Interestingly, Jurkat cell IL-10 and TGF-β1 secretion were reduced after exposure to different concentrations of aspirin. This suggests that aspirin can inhibit T cell function via the TIGIT-BCL2-BAX signaling pathway, reducing IL-10 and TGF-β1 secretion and improving effector T cell function, ultimately contributing to the inhibition of tumor progression. This is a novel molecular mechanism of CRC prevention by aspirin.

To confirm this molecular mechanism we utilized a mouse CRC model and investigated treatment with different doses of aspirin. Aspirin treatment resulted in remarkable reductions in tumor numbers, accompanied by significant reductions in spleen size. Slight changes in bodyweight were also evident in aspirin treated mice, and CD4+TIGIT+ T cell levels were significantly reduced by high-dose aspirin administration. TIGIT expression was detected on different Treg subsets. Compared with the AD group the levels of CD4+CD25+ T cells, CD4+CD25+FOXP3+ T cells, and CD4+CD25+FOXP3+TIGIT+ T cells were lower in the HA group. These observations demonstrated the potential benefit of clinical administration of aspirin for prevention of CRC. In the present *in vivo* study aspirin exhibited higher anti-tumor immunity effects with lower toxicity in comparison with the control group. These novel findings may have profound therapeutic implications with respect to the clinical application of aspirin. More molecular evidence is required however, to facilitate a comprehensive understanding of the appropriate application of aspirin for CRC in routine clinical practice.

## Conclusion

In summary, the current study yielded new evidence that aspirin could inhibit effector T cell function via the TIGIT-BCL2-BAX signaling pathway, reducing IL-10 and TGF-β1 secretion and improving effector T cell function, ultimately contributing to the inhibition of tumor progression. This is a novel molecular mechanism of CRC prevention by aspirin.

## Abbreviations

TIGIT: T cell immunoglobulin and ITAM domain
CRC: Colorectal cancer
ASA: Aspirin
Tregs: Regulatory T cells
FOXP3: forkhead box P3
GAPDH: glyceraldehyde 3-phosphate dehydrogenase
BCL2: B cell lymphoma 2
BAX: BCL2-associated X protein
ELISA: Enzyme-linked immunosorbent assay
IL: Interleukin
TGF: transforming growth factor
